# Structure guided deformable image registration for treatment planning CT and post stereotactic body radiation therapy (SBRT) Primovist^®^ (Gd‐EOB‐DTPA) enhanced MRI

**DOI:** 10.1002/acm2.12773

**Published:** 2019-11-22

**Authors:** Svetlana Kuznetsova, Petra Grendarova, Soumyajit Roy, Rishi Sinha, Kundan Thind, Nicolas Ploquin

**Affiliations:** ^1^ Department of Physics and Astronomy University of Calgary Calgary Alberta Canada; ^2^ Department of Oncology University of Calgary Calgary Alberta Canada; ^3^ Department of Radiation Oncology The Ottawa Hospital Cancer Program University of Ottawa Ottawa Ontario Canada; ^4^ Department of Medical Physics Tom Baker Cancer Centre Calgary Alberta Canada

**Keywords:** deformable image registration, liver, Primovist^®^, stereotactic body radiation therapy (SBRT), Velocity AI

## Abstract

The purpose of this study was to assess the performance of structure‐guided deformable image registration (SG‐DIR) relative to rigid registration and DIR using TG‐132 recommendations. This assessment was performed for image registration of treatment planning computed tomography (CT) and magnetic resonance imaging (MRI) scans with Primovist^®^ contrast agent acquired post stereotactic body radiation therapy (SBRT). SBRT treatment planning CT scans and posttreatment Primovist^®^ MRI scans were obtained for 14 patients. The liver was delineated on both sets of images and matching anatomical landmarks were chosen by a radiation oncologist. Rigid registration, DIR, and two types of SG‐DIR (using liver contours only; and using liver structures along with anatomical landmarks) were performed for each set of scans. TG‐132 recommended metrics were estimated which included Dice Similarity Coefficient (DSC), Mean Distance to Agreement (MDA), Target Registration Error (TRE), and Jacobian determinant. Statistical analysis was performed using Wilcoxon Signed Rank test. The median (range) DSC for rigid registration was 0.88 (0.77–0.89), 0.89 (0.81–0.93) for DIR, and 0.90 (0.86–0.94) for both types of SG‐DIR tested in this study. The median MDA was 4.8 mm (3.7–6.8 mm) for rigid registration, 3.4 mm (2.4–8.7 mm) for DIR, 3.2 mm (2.0–5.2 mm) for SG‐DIR where liver structures were used to guide the registration, and 2.8 mm (2.1–4.2 mm) for the SG‐DIR where liver structures and anatomical landmarks were used to guide the registration. The median TRE for rigid registration was 7.2 mm (0.5–23 mm), 6.8 mm (0.7–30.7 mm) for DIR, 6.1 mm (1.1–20.5 mm) for the SG‐DIR guided by only the liver structures, and 4.1 mm (0.8–19.7 mm) for SG‐DIR guided by liver contours and anatomical landmarks. The SG‐DIR shows higher liver conformality as per TG‐132 metrics and lowest TRE compared to rigid registration and DIR in Velocity AI software for the purpose of registering treatment planning CT and post‐SBRT MRI for the liver region. It was found that TRE decreases when liver contours and corresponding anatomical landmarks guide SG‐DIR.

## 1 INTRODUCTION

Stereotactic Body Radiation Therapy (SBRT) is an ablative technique characterized by the high dose delivered in one to five fractions with either the same or greater biologically effective dose as conventional radiotherapy.^1^ Studies have shown appreciable local control with the use of SBRT in primary and metastatic hepatic malignancies.^2^, ^3^, ^4^, ^5^, ^6^, ^7^, ^8^ SBRT has become the selected treatment of choice for patients who are not candidates for surgery due to tumor location.^9^ High dose per fraction and steep dose gradient is characteristic of SBRT treatments, which can lead to nontarget liver damage and potential hepatic toxicities that become limiting factors for optimal target dose delivery.^10^ This can have a significant adverse impact on patients' quality of life, and therefore assessment of liver function pre‐ and post‐SBRT treatment is essential.^1^

Child‐Pugh score is often utilized in order to evaluate the liver dysfunction using several markers of liver injury including biochemical and clinical, however the use as a predictor for the risk of radiation‐induced liver damage is arbitrary and somewhat limited as it does not provide any regional‐volumetric information on compromised liver function. Gadolinium‐ethoxybenzyl‐diethylenetriamine pentaacetic acid (Gd‐EOB‐DTPA) is an MRI contrast agent also known as Primovist^®^ (Bayer Pharma AG, Berlin, Germany) that is taken up by hepatocytes, and as a result the assessment of the intensity of liver parenchyma can be correlated to the liver dysfunction.^11^ Following contrast administration, Primovist^®^ is distributed into the vascular space during arterial and portal venous phases; subsequently it is taken up by the hepatocytes during the hepatobiliary phase, where an enhancement can be observed on delayed images. Liver parenchyma can then be correlated to the liver dysfunction based on decreased signal intensity.^12^

Thus, the Primovist^®^ contrast‐enhanced MRI scans contain the regional information pertaining to hepatic function. This contrast agent has been utilized for the assessment of liver dysfunction,^13^, ^14^, ^15^, ^16^, ^17^, ^18^, ^19^ as well as the quantification of the threshold dose for radiation‐induced liver reaction or hepatic toxicity.^11^, ^12^, ^20^, ^21^, ^22^, ^23^, ^24^ In order to determine the threshold dose, image registration is typically employed in order to obtain the intensity information from the MRI and dose information from planning CT for each liver voxel. Primovist^®^ MR imaging has proven itself to be a useful tool for liver function assessment. In order to properly determine the threshold dose associated with focal liver reaction, proper image registration needs to be implemented between planning CT which contains dose information and Primovist^®^ MRI scans.

Image registration consists of applying a transformation to the coordinate system that corresponds to the *source* image in order for it to be expressed in terms of *target* image's coordinate system. In the context of our study, registration of planning CT and post‐SBRT MRI can be difficult to perform due to the following reasons, a) Two scans of interest can be obtained under different immobilization conditions such as use of abdominal compression for planning CT. Since liver tissue is relatively malleable and elastic, the presence of abdominal compression during planning CT (but not on MRI) can result in significant differences in the shape of the organ on corresponding scans. b) A significant difference in liver volume can arise between the beginning of treatment and after the SBRT treatment. This has been observed in previous studies,^25^, ^26^ where 2–6 months after SBRT liver volume decreased on average by approximately 20%. The significant volume change creates additional variance between scans which can further complicate the image registration. c) Intensity‐based DIR of multimodality scans such as MRI and CT is challenging due to the differences in the definition of grayscale values that pertain to the very nature of modalities.^25^

The rigid registration technique allows for the image volume to be translated in three dimensions and rotated along the three axes thus limiting it to the total of six degrees of freedom.^26^ Rigid registration cannot account for any volumetric changes; therefore it can underperform for structures such as liver. A study by *Yu Ji et al 2013* looked at the performance of rigid registration for respiration‐gated MRI and 4D‐CT in radiation therapy liver patients.^27^ Using anatomical landmarks on corresponding scans, it was found that the position of these landmarks post rigid registration was approximately 5 mm. It is important to note that in the study mentioned, both of the types of scans were obtained on the same day under similar conditions. Therefore, applying rigid registration in the scenario where scans are taken at the opposite ends of the treatment timeline and also under different acquisition conditions, one can only expect for these metrics to deteriorate in performance.

DIR can account for volumetric changes between scans, but it can also produce an unrealistic output where voxels move in a nonphysiological manner. Therefore, TG‐132 recommends qualitative assessment in addition to quantitative calculations for image registrations. Examples of sites where DIR has been used in the past include prostate, head and neck, lung, and liver.^28^ An important challenge in DIR occurs when a specific organ is deformed relative to adjacent structures. The alignment of this organ to a nearby structure could be compromised relative to the best solution for the entire image volume.

Structure‐guided DIR (SG‐DIR) is a hybrid registration technique in Velocity AI 3.2 software (Varian Medical Systems, Inc, Palo Alto, CA, USA). It allows for the matching of the structures of interest using the specified contours and implements a B‐spline transformation along with normalized mutual information similarity metric^1^. Ten percent of the weight of SG‐DIR is associated with the alignment of structures using the sum of squared differences for the corresponding points of structures, and 90% of the weight is associated with the registration process using mutual information. Contour‐based DIR has been used in the past, but to our knowledge, its implementation was limited to the anatomical structures prone to significant volume change such as bladder and rectum, which also lack in anatomical landmarks.^29^, ^30^, ^31^, ^32^ The purpose of this study was to compare the performance of SG‐DIR to common types of image registration techniques provided by Velocity AI for multimodality imaging in radiation therapy specifically for the liver structure in relation to recommendations by TG‐132.

Validating image registration can be challenging due to the absence of established reference or standards. Physical and deformable phantoms with implanted fiducials can be used for the validation of DIR, however it is difficult to construct a phantom that fully resembles the physical properties of the anatomical scan, and it is even more difficult to construct such a phantom in the evaluation of multimodality image registration. A Task Group Report titled *Use of image registration and fusion algorithms and techniques in radiotherapy: Report of the AAPM Radiation Therapy Committee Task Group No. 132* (TG‐132)^26^ provides several metrics along with suggested tolerances that can be used in the evaluation of the performance of DIR. These metrics include target registration error (TRE), mean distance to agreement (MDA), Dice Similarity Coefficient (DSC), and Jacobian determinant. In this study, we used the suggested metrics from TG‐132 to evaluate the performance of SG‐DIR against rigid registration and DIR in Velocity AI. Furthermore, we assessed and compared different approaches to using SG‐DIR by guiding it using liver contours (SG‐DIR_liver_) and anatomical landmarks along with liver contours (SG‐DIR_liver+landmarks_). In addition, for selected set of patient scans, separate liver segments were contoured by a Radiation Oncologist in order to compare the regional performance of the image registration methods. To our knowledge, this is the first study to quantify the accuracy of two structure‐based DIR methods specifically applied to the planning CT and post‐SBRT MRI.

## 2 MATERIALS AND METHODS

### 2.1 SBRT Technique

Patients with liver tumors were enrolled in the study if they were suitable for liver SBRT, as well as for MRI acquisition. During planning 4D‐CT acquisition, patients were immobilized using a compression bridge. The internal target volume (ITV) was defined on combining gross tumour volumes on inspiration and expiration 4D‐CT sequences. Planning target volume (PTV) margin was defined as a 5mm isotropic extension on ITV. Average planning CT was used for treatment planning. For *Child‐Pugh A* score patients, the standard dose prescription was 50 Gy in five fractions, and 30–35 Gy in five fractions for *Child‐Pugh B* score patients. When critical structure constraints could not be achieved based on the standard prescription, total dose was lowered until the dose constraints of all critical structures were met. Target coverage criteria were comprised of 100% of prescription dose to at least 95% of the PTV. Treatment planning was VMAT‐based (Volumetric Modulated Arc Therapy) with 6FFF energy and treatment delivery was performed on the TrueBeam^TM^ Linac (Varian Medical Systems, Inc, Palo Alto, CA, USA), with CBCT as verification imaging, which was acquired prior to each fraction.

### 2.2 Image Registration

Each patient enrolled in the study, had a planning 4D‐CT and a Primovist^®^ contrast‐enhanced MRI obtained approximately 8–12 weeks post‐SBRT treatment. Planning CTs were obtained using a Philips^TM^ Brilliance Big Bore scanner with 1.37 mm pixel spacing and 3 mm slice thickness. MRI scans were obtained using GE Medical Systems^TM^ Optima MR360 with pixel spacing ranging from 0.70–0.90 mm and slice thickness of 2.5 mm. The liver was contoured on planning CT and MRI, and all of the contours were verified by a radiation oncologist. In addition, a radiation oncologist chose additional anatomical landmarks on both sets of scans (6–11 landmarks per patient); primarily vessel bifurcation, stents and calcifications if present.

Image registrations were performed in Velocity AI 3.2 software (Varian Medical Systems, Inc, Palo Alto, CA, USA). Initially, rigid registration was performed which implements normalized mutual information similarity metric. The original rigid registration was used as a baseline for further DIR; Rigid registration is typically required prior to implementation of DIR or SG‐DIR as it allows for the initial global alignment of two image volumes. Additionally, the region of interest (ROI) was established for DIR where it was large enough to encompass an entire liver volume. The SG‐DIR was performed with two different methods: the first approach, SG‐DIR was performed with the input being only liver contours on corresponding scans to guide the registration (SG‐DIR_liver_); the second approach was performed using liver contours and anatomical landmarks defined on MRI and CT where anatomical landmarks were treated as structures and their centers were overlaid to guide the registration (SG‐DIR_liver+landmarks_). Planning CT was registered to the post‐SBRT Primovist MRI that closely corresponded to the contrast phase of the CT, thus allowing for better intensity correspondence between scans, specifically that of the liver vessels, which helps better guide image registration. Both types of SG‐DIR used initial rigid registration as a baseline, and all types of DIR implemented B‐spline deformation with normalized mutual information similarity metric. Following the DIR and SG‐DIR, a displacement vector field (DVF) was obtained which contains the information of the movement of each voxel in the primary image with respect to the secondary. The DVF was used to assess the movement of voxels which specifically pertain to the liver structure. The DVF was exported from Velocity AI along with the planning CT, and liver structures were used as inputs for the custom written program in MATLAB R2016a. The program extracted the voxels that corresponded only to the liver structure, and then resampled the DVF in terms of the resolution of the planning CT and assigned the associated displacement magnitude for the each voxel within the liver structure. The cumulative histograms were constructed for the DIR and two types of SG‐DIR tested in this study in order to assess the overall movement of the liver due to image registration.

The following metrics from TG‐132 were used for image registration performance assessment: Dice Similarity Coefficient (DSC), Mean Distance to Agreement (MDA), Target Registration Error (TRE), and Jacobian determinant. DSC is defined as the volumetric overlap between two structures,

(1) $$\text{DSC}\left(A,B\right)=\frac{2\left|A\cap B\right|}{\left|A\right|+\left|B\right|}.$$

In the above equation, *A* and *B* represent two different liver structure volumes. MDA (also referred to as Mean Surface Distance) is defined by the computed minimum distance between surface points from liver structure *B* to liver structure *A*, where all of the distances are averaged.^26^ TRE is defined as the distance between locations of anatomical landmarks post registration, and is calculated as follows:

(2) $$\text{TRE}=\sqrt{\Delta x^{2}+\Delta y^{2}+\Delta z^{2}}.$$

Jacobian determinant corresponds to the voxel volume change following the DIR.^26^, ^34^ It is quantified using the DVF, where a vector for a voxel *i,* (**u_i_**=(u_xi_, u_yi,_ u_zi_), is used to create a Jacobian matrix from which a determinant is calculated:

(3) $$J_{i}=\left|\begin{array}{ccc} \frac{\partial u_{\mathrm{xi}}}{\partial x} & \frac{\partial u_{\mathrm{xi}}}{\partial y} & \frac{\partial u_{\mathrm{xi}}}{\partial z}\\ \frac{\partial u_{\mathrm{yi}}}{\partial x} & \frac{\partial u_{\mathrm{yi}}}{\partial y} & \frac{\partial u_{\mathrm{yi}}}{\partial z}\\ \frac{\partial u_{\mathrm{zi}}}{\partial x} & \frac{\partial u_{\mathrm{zi}}}{\partial y} & \frac{\partial u_{\mathrm{zi}}}{\partial z} \end{array}\right|.$$

Using the above approach, the Jacobian is calculated for every voxel thus creating a Jacobian map. ***J*** *_i_* greater than 1 corresponds to the increase in volume, ***J*** *_i_* less than 1 implies a decrease in volume, ***J*** *_i_* = 1 corresponds to no volume change, and ***J*** *_i_* < 0 is indicative of unrealistic and nonphysiological voxel motion such as DVF tearing and folding.^33^ The percentage of liver volume with negative Jacobian values was quantified for DIR, and two types of SG‐DIR. This gave an insight into which image registration approach was prone to more error.

### 2.3 Liver Segmentation

We used a simplified indigenous version of Radiation Therapy and Oncology Group consensus guideline for liver segmentation.^34^ Instead of mapping eight liver segments individually, we created the following segments of liver: right superior hepatic segment, right inferior hepatic segment, left medial hepatic segment, and left lateral hepatic segment. In addition to these segments, we contoured the intra‐hepatic portion of the portal vein (PV) till the section where it's confluence into right and left branches, the proximal 5 mm of these branches were also delineated. We also delineated the intrahepatic portion of the inferior vena cava (IVC) starting from the section where it branches off to right (RHV), left (LHV) and middle hepatic veins (MHV) until it pierces the diaphragm. Proximal 5 mm of the RHV, LHV and MHV were contoured along with the IVC. For contouring the liver segments, we excluded the IVC, caudal lobe (segment I) and the gallbladder from the whole liver contour. Left and right lobe of liver were separated by a plane extending vertically through the gallbladder fossa and MHV. The right superior hepatic segment was created by combining segments VII and VIII, and the right inferior hepatic segment consisted of segment V and VI. On the left side, the left medial segment was delineated by combining segment IVA and segment IVB while the left lateral segment was delineated combining segment II and III.

### 2.4 Statistical Analysis

The nonparametric statistical tests were performed for comparison in performance of various registration techniques. The Wilcoxon Signed Rank test was implemented in SPSS Statistics v23.0 (IBM Corp, NY, USA) with a significance level set at 0.05. The power analysis was conducted using GPower 3.1 Software^35^, ^36^ for the purpose of establishing the required patient cohort and the number of anatomical landmarks needed. For the power of 0.9, it was calculated that the required number of anatomical landmarks needed to observe the difference of 2 mm is n = 47; for the DSC, to observe a difference of 0.03, the required number of cases is N = 14; for MDA the required number of cases to observe the difference of 1 mm was N = 14. The median and mean along with standard error of the mean (SEM) defined by σ/√n (standard deviation defined by ***σ***, and number of samples defined by *n*) was calculated for DSC, MDA, TRE, and voxel displacement.

## 3 RESULTS

**Figure 1:**
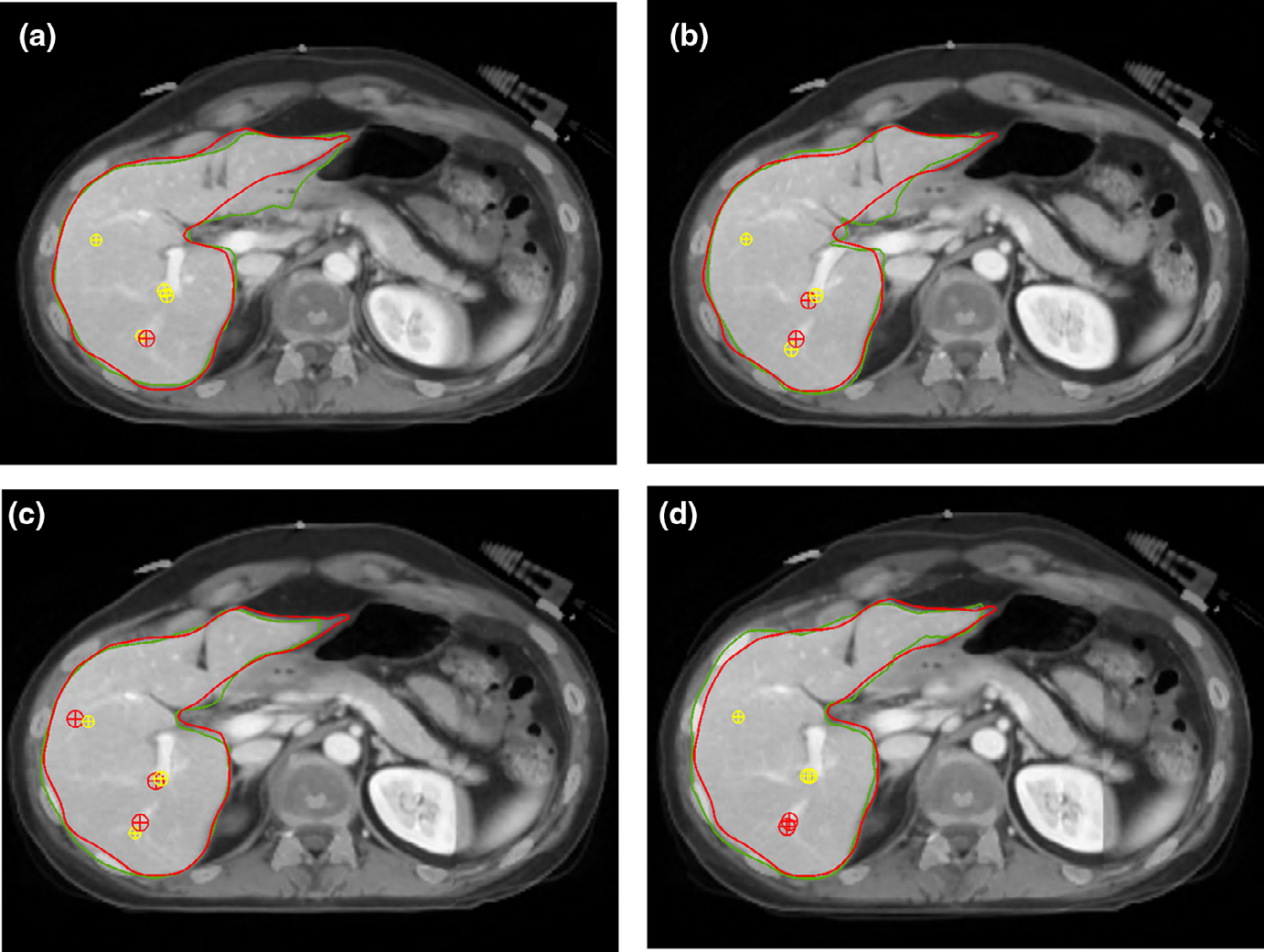
Planning CT and post‐SBRT MRI a) rigid registration b) DIR c)SG‐DIR_liver_, and d) SG‐DIR_liver+landmarks_. Corresponding landmarks are depicted by crosshairs, red contour corresponds to the liver structure on the planning CT, and green contour corresponds to the liver structure on the post‐SBRT MRI. SG‐DIR, structure‐guided deformable image registration.

**Figure 2:**
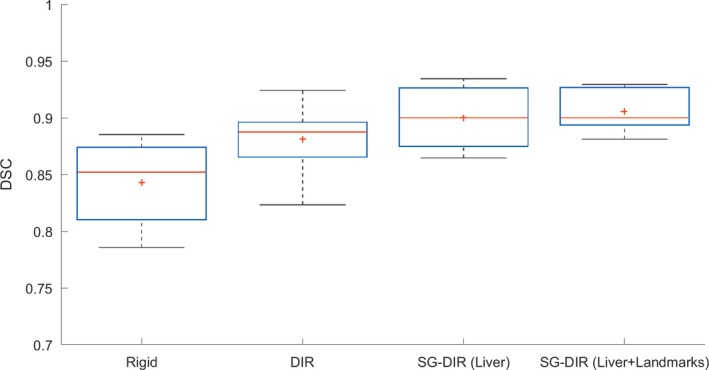
DSC results for the image registration methods for the 14 patient cohort. The solid red line corresponds to the median DSC and the red cross mark (+) corresponds to the mean DSC. DSC was quantified within Velocity AI software.

**Figure 3:**
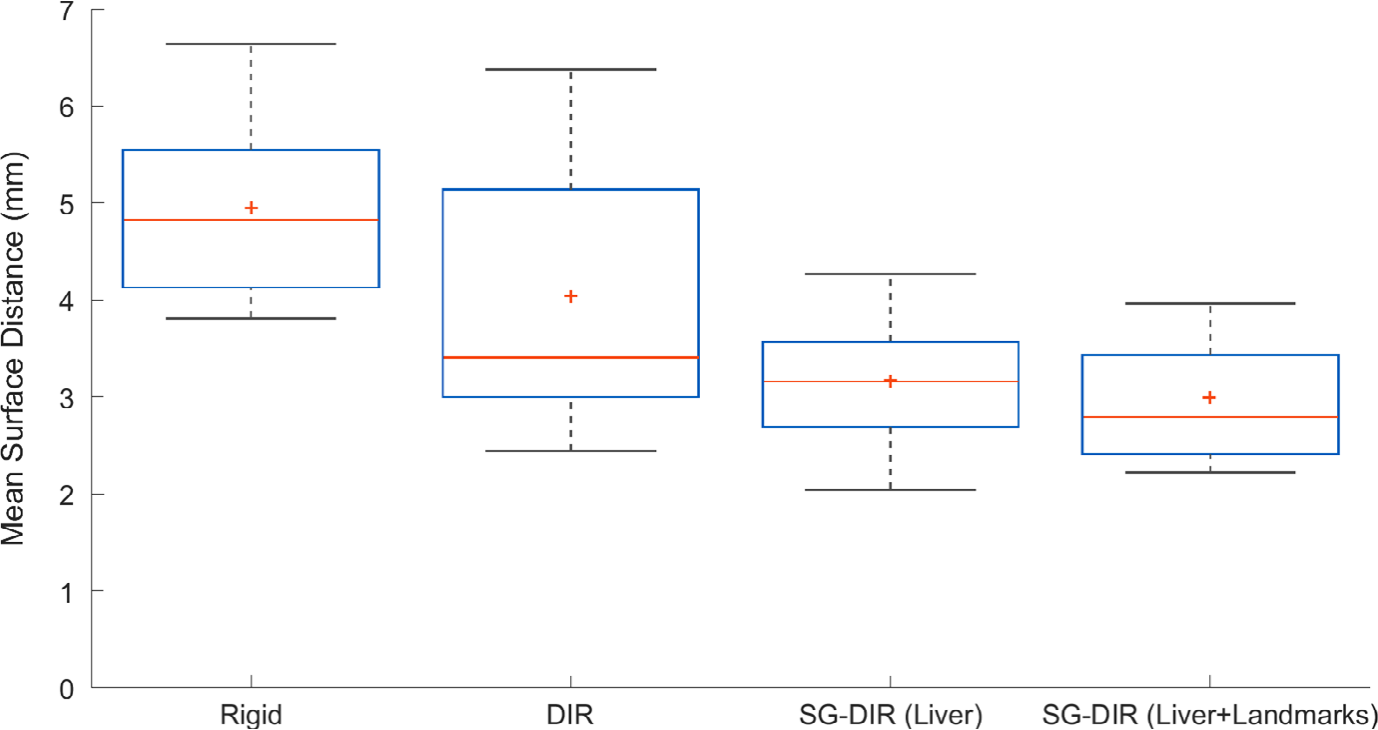
Mean surface distance (also known as *Mean Distance to Agreement (MDA)*) results for the image registration methods for the 14 patient cohort. The solid red line corresponds to the median MDA and the red cross mark (+) corresponds to the mean MDA. MDA was quantified within Velocity AI software.

An example of four image registration methods for one of the patients in the cohort is illustrated in Fig. 1. Rigid registration had the lowest DSC (mean ± SEM) of 0.84 ± 0.01, and both types of SG‐DIR had the highest DSC, 0.90 ± 0.01 and 0.91 ± 0.01. The DSC results are illustrated in Fig. 2, and the median with range is summarized in Table 1. The SG‐DIR_liver+landmarks_ had the lowest DSC range, while rigid registration and DIR had the highest DSC range. The MDA (mean ± SEM) for the 14 patient cohort was 4.9 ± 0.3 mm for rigid registration, 4.0 ± 0.5 mm for DIR, 3.2 ± 0.2 mm for SG‐DIR_liver_, and 3.0 ± 0.2 mm for SG‐DIR_liver+landmarks_. The MDA results are illustrated in Fig. 3 and are summarized in Table 1. There was a significant difference between the two types of SG‐DIR and rigid registration (*P* = 0.001), as well as between rigid and DIR (*P* = 0.005), and between SG‐DIR_liver+landmarks_ and DIR (*P* = 0.007) with respect to DSC. For MDA, there was a significant difference between rigid and two types of SG‐DIR (*P* = 0.001), and between DIR and SG‐DIR_liver+landmarks_ (*P* = 0.04).

**Table 1:** Median values with the corresponding range for Rigid, DIR, and two types of SG‐DIR methods.

Registration Type	Median DSC	DSC Range	Median MDA (mm)	MDA Range (mm)	Median TRE (mm)	TRE Range(mm)
Rigid	0.85	0.77–0.89	4.8	3.7–6.8	7.5	0.5–23
DIR	0.89	0.81–0.93	3.4	2.4–8.7	6.3	0.7–30.7
SG‐DIR (liver)	0.90	0.86–0.94	3.2	2.0–5.2	6.2	1.1–20.5
SG‐DIR (liver + landmarks)	0.90	0.87–0.93	2.8	2.1–4.2	4.3	0.8–19.7

DSC, dice similarity coefficient; MDA, mean distance to agreement; SG‐DIR, structure‐guided deformable image registration; TRE, target registration error.

**Figure 4:**
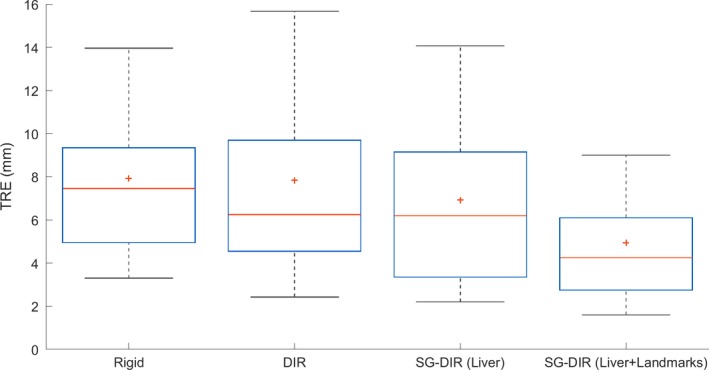
Target Registration Error (TRE) results for the image registration methods for 124 landmarks. The solid red line corresponds to the median TRE and the red cross mark (+) corresponds to the mean TRE. TRE was quantified within Velocity AI software.

The TRE (mean ± SEM) for the 124 anatomical landmarks acquired from the 14 patient cohort was 7.9 ± 0.4 mm for rigid registration, 7.8 ± 0.5 mm for the DIR, 6.9 ± 0.4 mm for SG‐DIR_liver_, and 4.9 ± 0.3 mm for SG‐DIR_liver+landmarks_. The TRE results for 124 landmarks acquired for 14 patients are illustrated in Fig. 4. There was a significant difference between SG‐DIR_liver+landmarks_ and DIR (*P* < 0.05), rigid registration (*P* < 0.05), as well as SG‐DIR_liver_ (*P* < 0.05).

There was also a significant difference between SG‐DIR_liver_ and rigid registration (*P* = 0.006).

The regional liver evaluation conducted for five patients has shown that SG‐DIR results in higher DSC for the Left Lateral segment, Left Medial segment, Right Lobe, Right Inferior segment, and Right Superior segment. Rigid registration resulted in higher median DSC for the portal vein (0.58), while DIR resulted in the highest median DSC for IVC (0.51). Table 2 shows the results for regional liver image registration performance for the four image registration methods tested in this study.

**Table 2:** Results for the five patients concerning the regional liver performance.

		Rigid	DIR	SG‐DIR (liver)	SG‐DIR (liver + landmarks)
IVC	Median DSC	0.45	0.51	0.38	0.43
	DSC Range	0.35–0.64	0.36–0.67	0.23–0.64	0.2–0.69
	Median MDA	3.9	3.9	4.47	5.2
	MDA Range	2.6–5.5	2.5–6.2	2.0–5.7	2.7–5.8
Portal Vein	Median DSC	0.58	0.64	0.45	0.46
	DSC Range	0.46–0.75	0.42–0.69	0.30–0.70	0.3–0.71
	Median MDA	2.0	2.8	3.9	3.7
	MDA Range	1.9–4.7	2–5.7	2.4–6.7	1.9–5.4
Left Lobe	Median DSC	0.8	0.81	0.81	0.85
	DSC Range	0.61–0.87	0.77–0.86	0.79–0.91	0.76–0.91
	Median MDA	4.1	3.8	3.6	3.3
	MDA Range	3–7.9	3.2–5	2.3–4.4	2.2–5.7
Left Lateral Segment	Median DSC	0.73	0.80	0.79	0.80
	DSC Range	0.58–0.86	0.7–0.85	0.76–0.88	0.7–0.89
	Median MDA	4.1	4.3	3.2	3.3
	MDA Range	3.6–8.1	3–5	2.9–4.8	2.7–6.6
Left Medial Segment	Median DSC	0.74	0.73	0.73	0.74
	DSC Range	0.46–0.77	0.56–0.75	0.57–0.82	0.57–0.82
	Median MDA	4.8	5.1	4.4	4.0
	MDA Range	3.8–7.4	3.7–5.8	3.6–6.5	3.6–5.9
Right Segment	Median DSC	0.86	0.87	0.88	0.90
	DSC Range	0.77–0.91	0.84–0.91	0.85–0.92	0.85–0.9
	Median MDA	4.4	4.1	3.7	3.2
	MDA Range	3.2–6.7	2.9–4.9	2.6–4.5	2.7–4.5
Right Inferior Segment	Median DSC	0.79	0.83	0.84	0.84
	DSC Range	0.77–0.91	0.71–0.89	0.78–0.87	0.8–0.89
	Median MDA	4.2	3.5	3.2	3.5
	MDA Range	1.9–5.6	2.5–6.7	2.9–5	2.3–4.5
Right Superior Segment	Median DSC	0.81	0.82	0.86	0.86
	DSC Range	0.67–0.88	0.71–0.88	0.78–0.87	0.78–0.88
	Median MDA	4.6	5.1	4.3	4.0
	MDA Range	3.8–6.9	2.8–6.6	3.3–5	3.2–5.2

DSC, dice similarity coefficient; IVC, inferior vena cava; MDA, mean distance to agreement; SG‐DIR, structure‐guided deformable image registration; TRE, target registration error.

**Figure 5:**
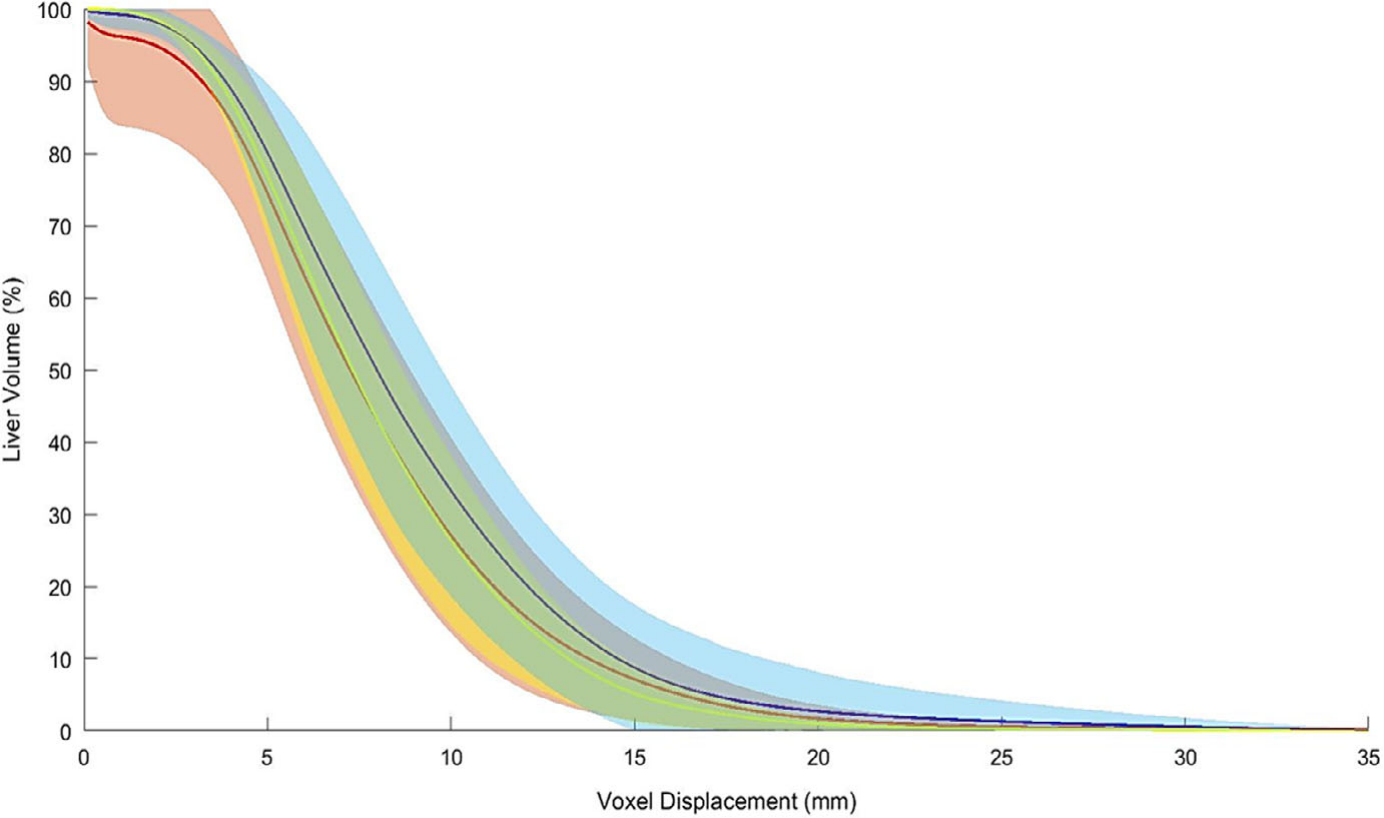
Cumulative histogram for the voxels within the liver structure for the DIR and two types of SG‐DIR. The solid line corresponds to the average liver volume, and the shaded transparent region is the standard error of the mean (SEM) for the 14 patients. SEM is defined by ***σ/√n***, where ***n*** corresponds to the number of samples, and ***σ*** corresponds to the standard deviation.

The output of the custom written program concerning the voxel displacement for DIR, SG‐DIR_liver_, and SG‐DIR_liver+landmarks_ is illustrated in Fig. 5. The two types of SG‐DIR methods had lower mean voxel displacement compared to DIR; 7.7 mm (SEM = 0.003 mm) and 7.9 mm (SEM = 0.002 mm) for SG‐DIR_liver_ and SG‐DIR_liver+landmarks_ respectively, and 9.0 mm (SEM = 0.003) for DIR evaluated for 14 patients.

Jacobian determinant analysis was performed for each liver structure on the 14 patients within Velocity AI software. For the DIR approach, only two out of 14 cases had 2.3% of the liver volume with negative Jacobian determinant values (median = 0); all other cases did not have any voxels that contained negative Jacobian determinant values. The SG‐DIR_liver_ method had relatively the highest median error where 0.56% (0–3.6%) of liver volume was below 0. The SG‐DIR_liver+landmarks_ method had a reduction in the liver percentage of negative Jacobian determinant values with the median percentage being 0.17% (0–2.3%). In 11 cases, there was a reduction in the liver volume with negative Jacobian value for SG‐DIR_liver+landmarks_ method. For one of the patients the number of voxels reduced from 3.6% to 0.1% when the SG‐DIR_liver+landmarks_ method was implemented.

## 4 DISCUSSION

According to TG‐132 recommendations, all four image registration approaches fall within DSC recommendations (~0.8–0.9), however the recommendations concerning MDA are only met by the SG‐DIR_liver+landmarks_ method. The trend observed for MDA and DSC agree for liver conformality; specifically, for the lowest DSC there is highest MDA, confirming the consistency in measurement. Additional constraints on the registration that come in the form of structures and landmarks result in a more consistent image registration where the MDA is minimized and DSC is maximized within the cohort (Table 1, Fig. 2).

According to Fig. 4 and Table 1, the smallest median TRE of 4.3 mm was observed for SG‐DIR_liver+landmarks_, which was statistically different from SG‐DIR_liver_. It is important to note that neither of the image registration methods assessed in this study met the TG‐132 recommendations for TRE of (~2–3 mm); this likely results from the inherently challenging image registration scenario where immobilization and volumetric changes caused by the dose deposition are present. Select past studies that looked at the CT‐MRI liver region registration according to the MIDRAS (Results of a **M**ulti‐**I**nstituion **D**eformable **R**egistration **A**ccuracy **S**tudy) have found that the average error ranged from 3.9 to 6.5 mm for different registration approaches.^37^ More recently, the study by *Manuel et al. 2014* used an organ‐focused mutual information approach for the purpose of registering CT and Gd‐EOB‐DTPA MRI has reported a median landmark‐based error of 6.86 mm, and a median surface‐based mean error of 3.23 mm.^38^ Furthermore, the majority of selected studies tended to acquire images on the same day,^27^, ^39^, ^40^ or in the case of *Manuel et al. 2014*, the MRI scans did not correspond to patients who had undergone radiation therapy. The use of anatomical landmarks as structures in the SG‐DIR approach has shown to significantly reduce the TRE as well as reduce error in the image registration as was shown through the quantification of Jacobian determinant.

Surprisingly the Jacobian determinant analysis with the focus on negative values, has shown that there is minimal error associated with DIR as opposed to SG‐DIR. It also appears that SG‐DIR_liver_ results in higher volumetric changes of voxels as opposed to DIR. The opposite was observed for the magnitude of displacement of voxels, where DIR had a higher overall voxel displacement compared to SG‐DIR, as depicted in Fig. 5. These observations are indicative of the nature of the image registration behavior of SG‐DIR versus DIR; specifically, structures in SG‐DIR restrain the movement of voxels thus reducing the overall displacement magnitude, and as a result the voxel‐based volumetric expansion/contraction is more likely to occur. The DIR on the other hand has a much larger range of voxel displacement, and as a result the magnitude of volumetric expansion/contraction of voxels is reduced. It is difficult to state with certainty the nature of SG‐DIR versus DIR methods, however the voxel displacement and Jacobian Determinant can give an insight into their relative expected behaviors.

Looking closer at the performance of DIR for the individual liver segments (Table 2), we found that SG‐DIR underperforms for two out of eight structures delineated: IVC and portal vein. A previous study that looked at the image registration of the liver for noncontrast‐enhanced planning CT and post‐SBRT MRI using regional liver segment analysis has found that the delineated portal region and combined IVC with caudate lobe had the lowest DSC compared to other segments such as the left lobe.^41^ Our results agree with the study, as well as illustrate the fact that having contrast‐enhanced scans does not improve the registration performance of the portal region and IVC. The underperformance of SG‐DIR for these cases could be the result of the fact that 90% of the weight of the algorithm is intensity based^2^. The percentage of the PTV volume encompassed by each liver segment was quantified; it was found that a large portion of the PTV tended to reside in the right hepatic lobe with the median PTV volume of 63% (31–100%). The right hepatic lobe had the lowest median MDA (3.2 mm) and highest median DSC (0.9) when SG‐DIR_liver+landmarks_ was utilized. Proper registration of the PTV region becomes vital since the corresponding PTV area on the post‐SBRT Primovist^®^ MRI is susceptible to decrease in the intensity signal. The results suggest that the SG‐DIR_liver+landmarks_ approach results in a higher segment conformality compared to other image registration methods for the right hepatic lobe which tends to contain the majority of the target's volume. Due to a small subset of patients, the regional liver assessment of the image registration performance is limited in terms of its significance. This analysis was used to better evaluate the relative behavior within the liver region post image registration in terms of qualitative analysis rather than quantitative.

It is important to note some of the limitations of the study, specifically the fact that the inter‐ and intra‐observer variability was not quantified. Several previous studies have been conducted where intra‐ and inter‐observer variability has been quantified for the delineation of the liver. The intra‐observer variability was quantified to be 0.96–0.98 and 0.95–0.98 for the inter‐observer variability using DSC formalism.^42^, ^43^, ^44^, ^45^ The inter‐observer variability for liver delineation is expected to be low since the edge information of the organ is typically very well defined on both CT and MRI. Furthermore, we were interested in assessing the performance of SG‐DIR with respect to other common types of image registration such as rigid registration and DIR. In addition this study had a small cohort, which does not take into account different types of tumor and liver cirrhosis conditions.

Our study used operator‐dependent strategies, which are dependent on manual contouring, contour propagation, and landmark identification. As summarized by *Paganelli et al 2018,* this is time consuming and operator‐dependent approach for geometric validation of image registration.^46^ There is a need for automatic implementation of contour delineation and anatomical landmark localization in order to help make patient‐specific DIR validation more routine in practice. Automatic extraction of landmark identification for multimodality images (CT and MRI) has so far been limited to only 2D cases, and stands to be a challenge.^46^ Better automating the auto‐segmentation and landmark extraction methods will allow for easier adaptation of DIR in a clinical setting, where performance assessment of image registration remains a limiting factor of DIR utilization.

## 5 CONCLUSION

This study evaluated the performance of SG‐DIR for the liver structure and its internal segments with respect to rigid registration and DIR in Velocity AI software. The SG‐DIR approach resulted in highest liver conformality and lowest TRE; it was found that the use of SG‐DIR that involves liver contours along with anatomical landmarks significantly reduces the TRE. The implementation of SG‐DIR requires more of a demanding workflow consisting of structure delineation and possibly anatomical landmark selection. However, despite the additional steps required for the implementation of SG‐DIR, this process provides a superior registration in the challenging scenario of image registration of planning CT and post‐SBRT MRI.

## CONFLICT OF INTEREST

The authors of this publication have no conflict of interest to declare.

6

**Figure 6:**
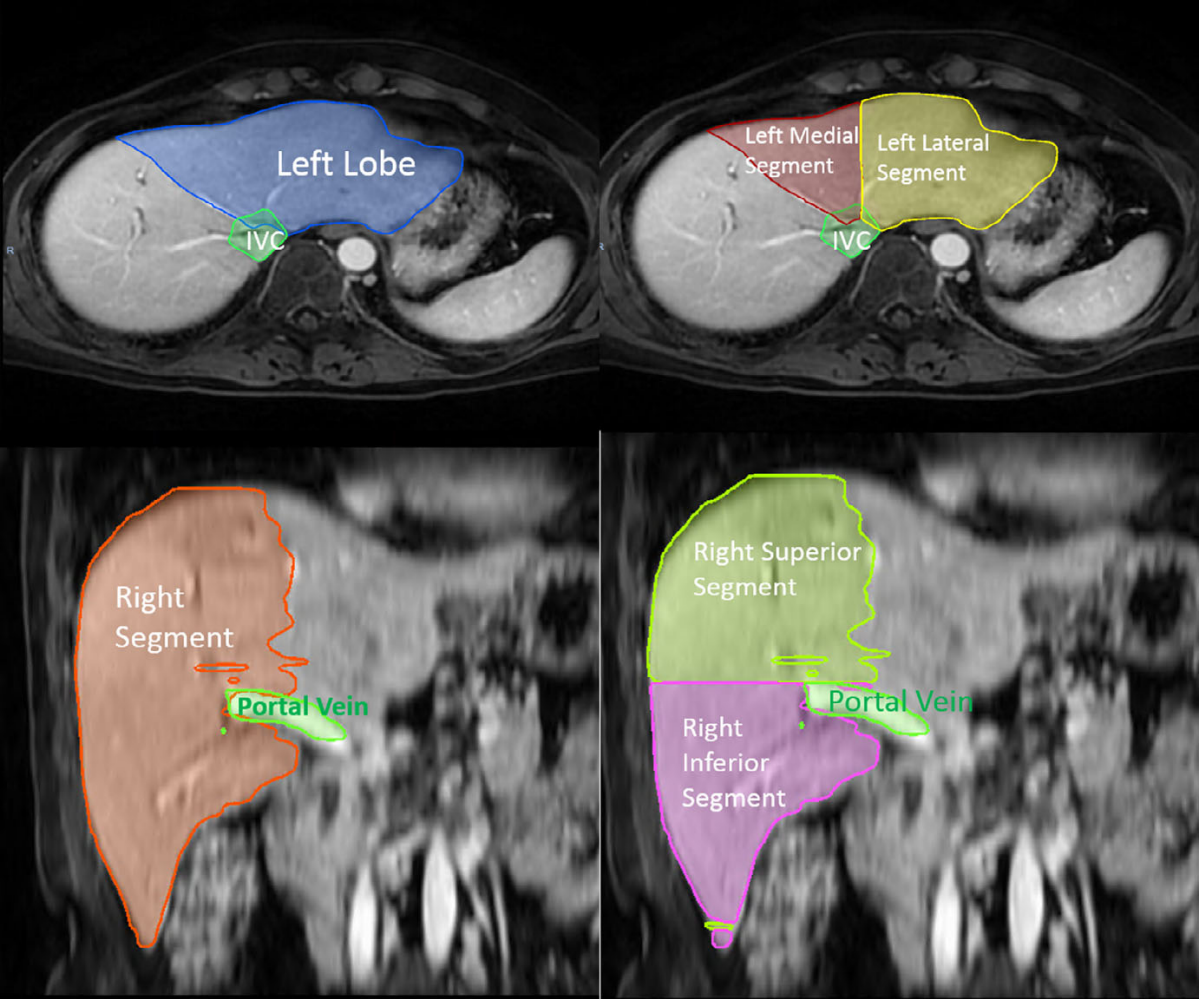
An example of one of the cases where separate hepatic segments were contoured. The contoured liver segments included IVC, left lobe, left medial segment, left lateral segment, right segment, right superior segment, right inferior segment, and portal vein. IVC, inferior vena cava.

## Notes

1 Velocity 3.2.0 User Manual. Varian Medical Systems, Inc. Atlanta GA, USA.

2 Velocity 3.2.0 User Manual. Varian Medical Systems, Inc. Atlanta GA, USA.
